# Inducible Knockdown of *Plasmodium* Gene Expression Using the *glmS* Ribozyme

**DOI:** 10.1371/journal.pone.0073783

**Published:** 2013-08-30

**Authors:** Parichat Prommana, Chairat Uthaipibull, Chayaphat Wongsombat, Sumalee Kamchonwongpaisan, Yongyuth Yuthavong, Ellen Knuepfer, Anthony A. Holder, Philip J. Shaw

**Affiliations:** 1 National Center for Genetic Engineering and Biotechnology (BIOTEC), Khlong Nueng, Khlong Luang, Pathum Thani, Thailand; 2 Division of Parasitology, MRC National Institute for Medical Research, London, United Kingdom; Liverpool School of Tropical Medicine, United Kingdom

## Abstract

Conventional reverse genetic approaches for study of *Plasmodium* malaria parasite gene function are limited, or not applicable. Hence, new inducible systems are needed. Here we describe a method to control *P. falciparum* gene expression in which target genes bearing a *glmS* ribozyme in the 3′ untranslated region are efficiently knocked down in transgenic *P. falciparum* parasites in response to glucosamine inducer. Using reporter genes, we show that the *glmS* ribozyme cleaves reporter mRNA *in vivo* leading to reduction in mRNA expression following glucosamine treatment. Glucosamine-induced ribozyme activation led to efficient reduction of reporter protein, which could be rapidly reversed by removing the inducer. The *glmS* ribozyme was validated as a reverse-genetic tool by integration into the essential gene and antifolate drug target dihydrofolate reductase-thymidylate synthase (*Pf*DHFR-TS). Glucosamine treatment of transgenic parasites led to rapid and efficient knockdown of *Pf*DHFR-TS mRNA and protein. *Pf*DHFR-TS knockdown led to a growth/arrest mutant phenotype and hypersensitivity to pyrimethamine. The *glmS* ribozyme may thus be a tool for study of essential genes in *P. falciparum* and other parasite species amenable to transfection.

## Introduction

Malaria remains a major global health problem, and eradication will not be possible until more is understood of *Plasmodium* parasite biology. Improved genetic technologies for the manipulation of *Plasmodium* are needed, in particular robust and scalable systems for conditional gene expression [1]. Genes with essential functions in blood stages cannot be knocked out conventionally to create null mutants owing to the haploid nature of the parasite, except in the special case where the gene function can be complemented chemically [2]. To overcome this limitation, conditional gene knockout using site-specific recombinases has been demonstrated. In this approach, transgenic parasites are generated which express recombinase in a controlled manner, either using a stage-specific promoter in the case of FLP/FRT-mediated excision [3], or by ligand activation for DiCre recombinase [4]. Cognate target sites for the recombinase are inserted into the gene of interest by homologous integration in such a way that the 3′ UTR sequence is excised by the recombinase. The control of recombinase in the DiCre system allows for conditional knockout of essential genes, as shown in the related Apicomplexan *T. gondii* [5]. Despite obtaining high efficiency of DiCre excision in *P. falciparum*, Collins *et al*. found that the level of target protein was unaffected owing to the use of an alternative transcription termination site [4]. This phenomenon has also been observed for FLP/FRT-mediated excision [6], and thus could limit the general usefulness of the inducible gene knockout strategy in *Plasmodium*.

In contrast to knockout for testing loss of gene function, methods are available for attenuating, or knocking-down gene expression. RNA interference, the most commonly used technique for attenuating expression in model organisms, is not applicable in *Plasmodium* species since they lack the required genes [7]. Recently, it has been shown that expression of essential *Plasmodium* genes can be attenuated using a tet-off system [8]. In this strategy, a transcription factor gene comprised of tet repressor and activating domain sequence (TRAD) is integrated upstream of the gene of interest. The target gene's promoter is replaced by a minimal promoter containing tet operator sequences (TetO), which are binding sites for the TRAD protein. The DNA binding activity of the TRAD protein is regulated by anhydrotetracycline (ATc), which can lead to highly efficient knockdown (up 95% inhibition) of the target gene in the murine malaria parasite *P. berghei* [8]. Control of episomal reporter gene transcription using the tet-off system has been demonstrated for *P. falciparum* [9]; however, control of endogenous *P. falciparum* genes by this method has, to our knowledge, not been reported. The tet-off system is currently impractical for controlling endogenous *P. falciparum* genes since the target gene must be modified by double cross-over integration of transgenic DNA. Double cross-over occurs at a very low frequency in *P. falciparum*, necessitating a lengthy negative selection step in transfection experiments [10]. The tet-off system also requires large transfection plasmids containing sequences homologous to the target gene 5′ flanking region. These 5′ flanking sequences in *P. falciparum* are typically very AT-rich and repetitive, and are often difficult to propagate in *Escherichia coli* when cloned in plasmids [11]. Until more robust methods other than cloning in plasmids are developed for making transfection DNA, the tet-off system will be experimentally challenging in *Plasmodium*.

Conditional loss of function phenotypes have been obtained in *P. falciparum* using the destabilizing domain (DD) system, in which protein stability is controlled by the Shld-1 ligand [12]. Significant down-regulation of essential parasite protein levels has so far only been demonstrated successfully for proteins expressed at low concentration [13] [14] [15], which is consistent with the lower efficiency of DD-mediated knockdown in *P. falciparum* compared with higher eukaryotes [12]. A recent study of DD efficiency in *P. falciparum* showed that the maximum difference achievable between ligand-stabilized and destabilized target protein levels is about five-fold [16]. Analysis of DD-mediated mutant phenotypes could be confounded by the observation that Shld-1 inhibits growth at 0.5 μM, the concentration needed to stabilize the target protein [13] [16]. An alternative DD of similar efficiency comprised of a mutated *E. coli* DHFR domain that is stabilized by trimethoprim has been demonstrated in *P. falciparum* [17]. This system has the advantage that the stabilizing ligand is much cheaper, although prior genetic modification of the parasite is required to make it resistant to trimethoprim, a moderately potent anti-malarial drug. The most important caveat of the DD approach limiting its use for large-scale reverse genetics is that the DD-fused protein may not function correctly, even when stabilized by ligand. The inability to integrate the DD sequence at many different *P. falciparum* loci has been attributed to this phenomenon [16].

As an alternative to protein regulation by DDs, conditional loss of gene function can be mediated at the mRNA level. Ribonuclease-P mediated cleavage of the *P. falciparum* essential *PfgyrA* mRNA has been achieved using an external guide sequence (EGS) RNA, with a loss of function phenotype demonstrated [18]. However, it is not yet clear how generally useful this strategy is, since off target effects have not been investigated. Moreover, the EGS must be delivered as a modified Morpholino oligonucleotide, the synthesis of which requires expensive and proprietary methods. Naturally occurring self-cleaving RNAs (ribozymes) are modular elements that retain function in different RNA contexts, and can be engineered to respond to ligands [19]. The Sm1 hammerhead ribozyme functions efficiently *in vivo* when placed in the context of a *P. falciparum* mRNA; however, non-toxic and specific inhibitors of this ribozyme could not be found, limiting its use as a reverse genetic tool [20].

**Figure 1:**
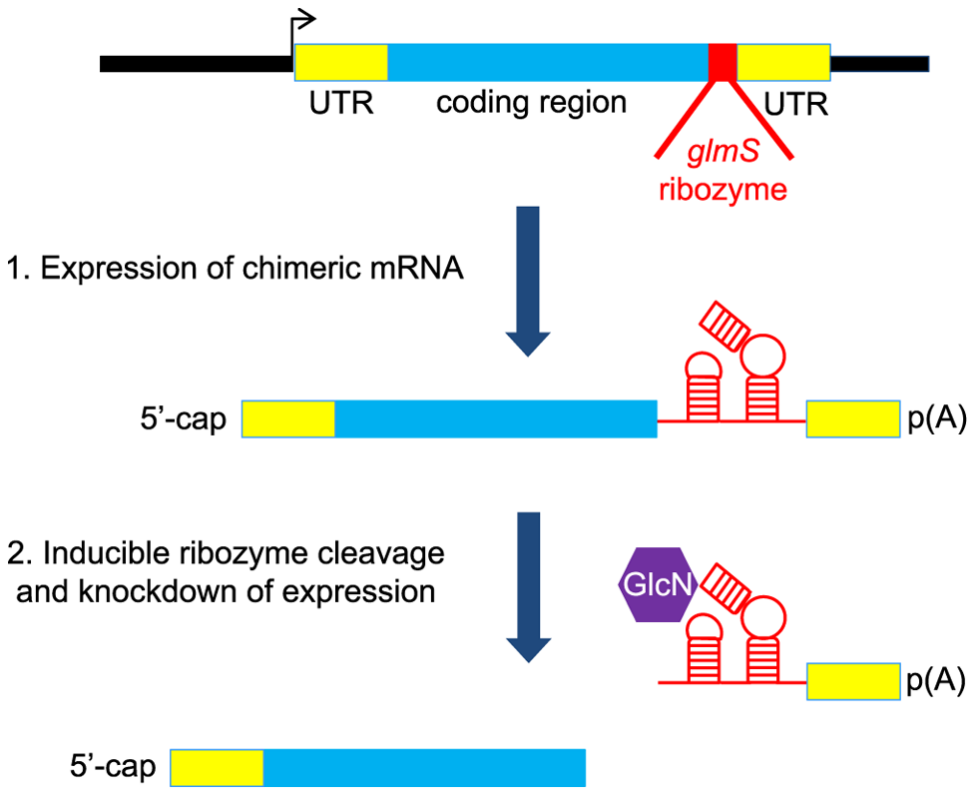
Schematic of the *glmS* ribozyme reverse genetic tool. The ribozyme is inserted in the 3′-UTR after the coding region so that it is present in the expressed mRNA. Following addition of the inducer, glucosamine, which binds to the ribozyme, the mRNA self-cleaves resulting in degradation of the mRNA and knock down of protein expression.

The *glmS* ribozyme from Gram-positive bacteria is unique since it has a specific co-factor requirement and has low activity during normal cell growth conditions when the co-factor is limiting [21]. Moreover, the *glmS* ribozyme has been shown to control reporter gene expression in *Saccharomyces cerevisiae* yeast in response to exogenous glucosamine (GlcN) [22]. Given these properties of the *glmS* ribozyme, we tested whether it could be used as a reverse-genetic tool in *P. falciparum*. In our system, insertion of the ribozyme sequence into a target gene leads to expression of chimeric *P. falciparum* target RNA with ribozyme RNA at the 3′ end within the UTR (Fig. 1). The degree of ribozyme self-cleavage, and thus the degree of target gene attenuation can be controlled by the concentration of GlcN added to the parasite culture medium.

## Materials and Methods

### Ethics statement

Human O+ erythrocytes and serum were obtained from healthy, non-pregnant volunteers aged 21–50 following the Thai Red Cross National Blood Center protocol. All volunteers were recruited from members of the public, and they completed and signed consent forms prior to blood donation. Volunteers were excluded from donation if they appeared unhealthy, or were during the course of medication, or had high blood pressure, or had had less than 8 hours sleep the night before donation. The consent forms and protocol for blood collection were approved by the BIOTEC Ethics Committee. After collection of blood, no records were made on containers which could link individual donors to blood donations.

### Construction of transfection plasmids

The pSSPF2/*Pf*Hsp60-GFP-Link plasmid described in [23] was used to construct transfection plasmids. Overlapping synthetic oligonucleotides corresponding to the wild-type and “M9” mutated *B. subtilis glmS* ribozyme sequences reported in [21] were combined in gene synthesis-PCR and the resulting 166 bp ribozyme element cloned at the 3′-UTR position downstream of the GFP gene in pSSPF2/*Pf*Hsp60-GFP-Link via the *Xho*I and *Pst*I sites. Oligonucleotides syn1-syn8 were used to construct wild-type *glmS* and oligonucleotides syn1-syn7 and syn9 were used to construct the M9 variant (Table S1).

**Figure 2:**
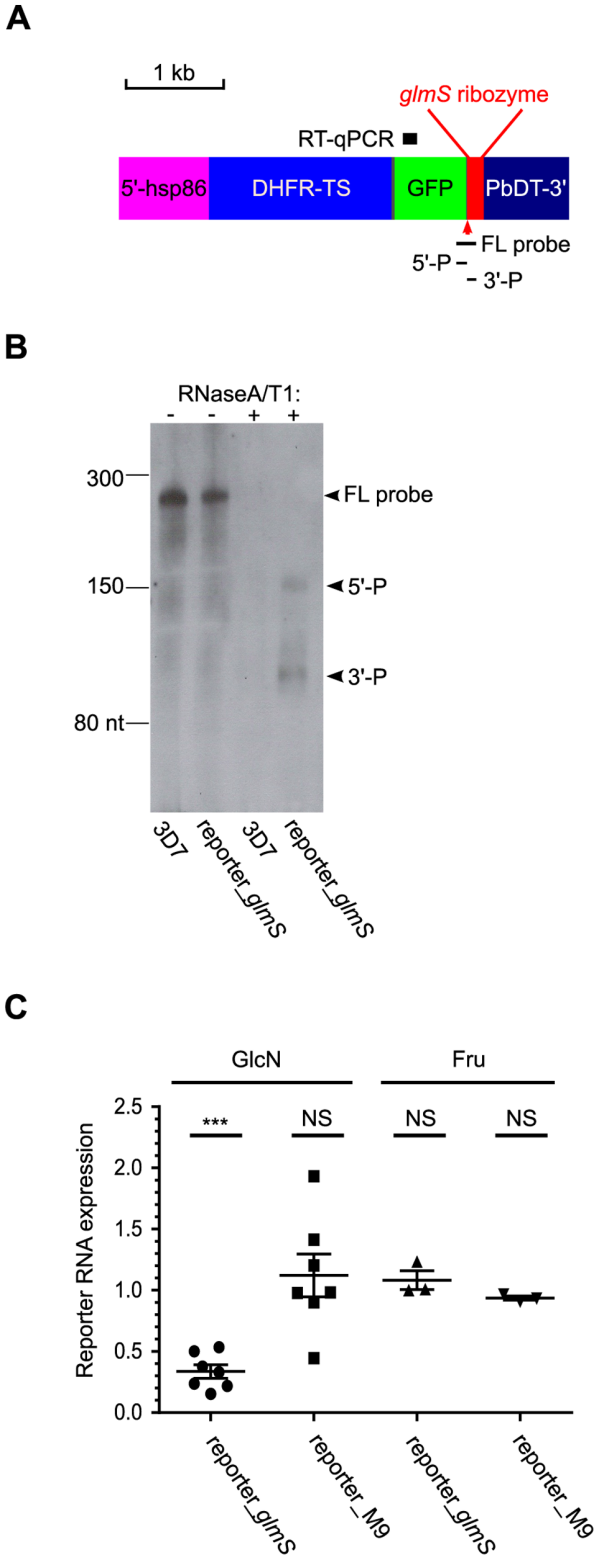
*glmS* ribozyme cleavage and control of *P. falciparum* mRNA expression. (A) Schematic diagram of the DHFR-TS-GFP reporter gene with *glmS* ribozyme in the 3′-UTR position (reporter_*glmS*). The reporter gene is flanked by 5′-hsp86 and PbDT-3′ *Plasmodium* transcriptional regulatory sequences. The sequence regions analyzed in parts B and C are marked: FL probe, antisense RNA probe for RNase protection assay; 5′-P and 3′-P, 5′ and 3′ ribozyme cleavage products, respectively; RT-qPCR, amplicon for RT-qPCR analysis of reporter mRNA levels. (B) RNase protection assay revealed *glmS* ribozyme cleavage products (arrowed as 5′-P and 3′-P respectively) in *P. falciparum* expressing reporter_*glmS*. 10% ring-stage synchronized parasites were treated for 24 h in the presence of 10 mM GlcN prior to harvesting and extraction of total parasite RNA. The 3D7 wild-type parasite was used as a control to test for probe specificity. Control hybridizations and gel analysis without RNase (lanes 1 and 2 marked as -) were also performed to demonstrate integrity of the RNA probe. The migration of the full-length RNA probe complementary to the *glmS* RNA (FL probe) and small RNA ladder (New England Biolabs) bands are marked. (C) Analysis of reporter mRNA expression in response to treatment with 10 mM GlcN and Fru. The expression levels of reporter_*glmS* mRNA or reporter_M9 mRNA (bearing inactivating mutations in the *glmS* ribozyme cleavage site) in treated cultures relative to untreated were determined from RT-qPCR using the ΔΔCq method normalized to BSD mRNA. Starting with 10% ring-stage synchronized cultures, parasites were treated with 10 mM GlcN or Fru for 24 h prior to harvesting and RNA extraction. Error bars represent S.E.M. (*n* = 7 for GlcN experiments, *n* = 3 for Fru experiments). One sample two-tailed *t*-tests were performed to determine if change in mRNA expression was significant; NS denotes not significant, *** denotes highly significant. The calculated *P*-values comparing sample means against hypothetical mean = 1 were: reporter_*glmS* GlcN treatment, <0.0001; reporter_M9 GlcN treatment, 0.5136; reporter_*glmS* Fru treatment, 0.3986; reporter_M9 Fru treatment, 0.0619.

**Figure 3:**
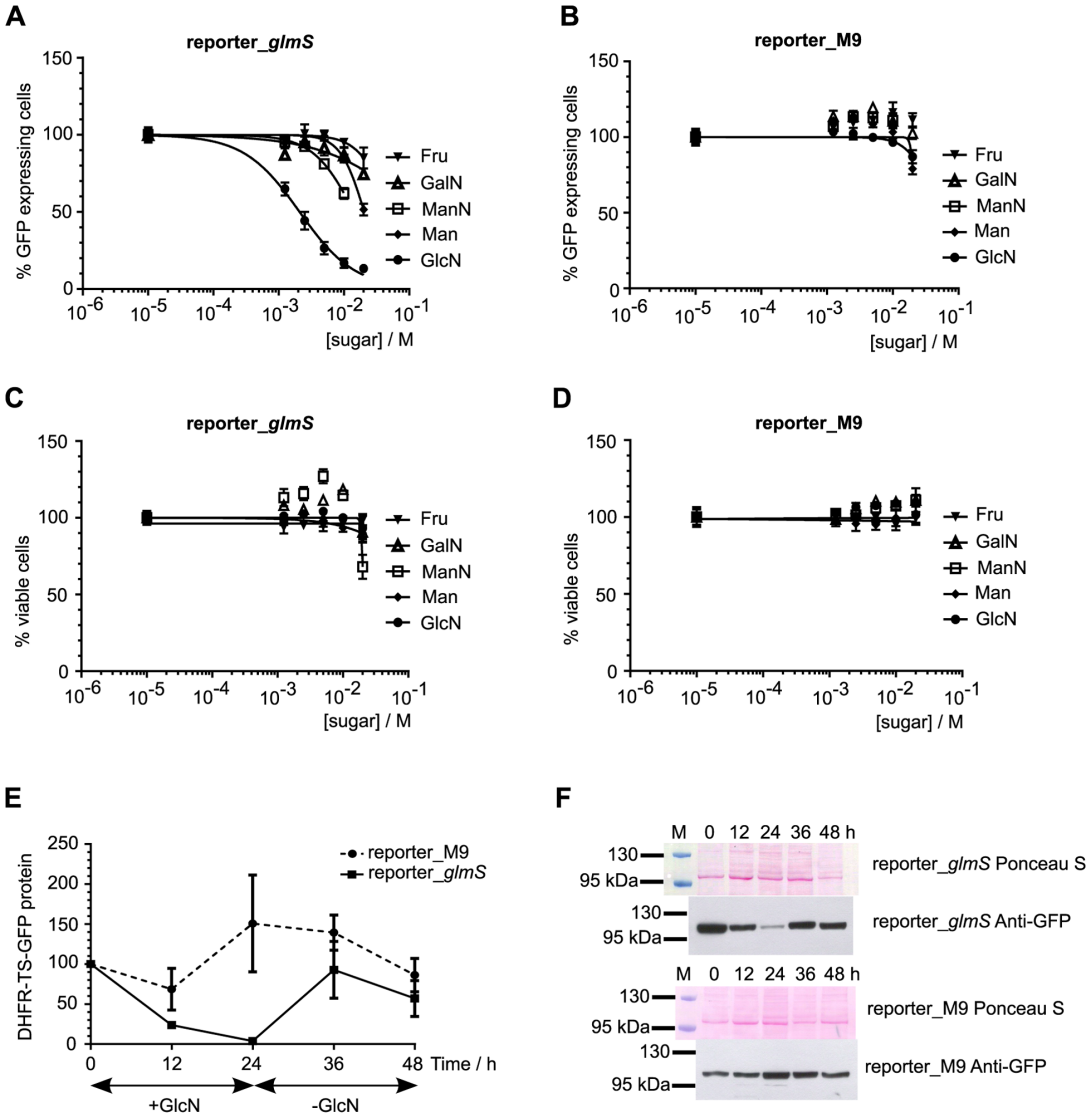
Ribozyme-mediated knockdown of reporter protein expression in response to sugar treatment. Ring-stage synchronized parasites from reporter_*glmS* (parts A, C) and reporter_M9 (parts B, D) transfected lines were treated for 24 h with varying concentrations of different sugars (GlcN, ManN, GalN, Fru and Man) prior to flow cytometric analysis. The numbers of GFP positive and viable cells were normalized to untreated controls, which were set as 100%. Data are the mean from triplicate experiments and error bars represent S.E.M. Part E shows that ribozyme-mediated knockdown of reporter protein is reversible. 10% ring-stage parasites were treated with 10 mM GlcN for 24 h and samples taken after 12 and 24 h of treatment. The parasite culture medium was changed and the culture was continued, with samples taken after 12 and 24 h in the new GlcN-free medium. The experiment was performed in triplicate with independent cultures for each replicate (5 sampled time points from each culture). Western immunoblot analysis of DHFR-TS-GFP reporter protein expression was performed using an anti-GFP polyclonal antibody. The normalized intensity of the ∼100 kDa band corresponding to DHFR-TS-GFP protein was measured relative to the normalized intensity at 0 h, which was taken as 100%. Data are the mean and error bars represent S.E.M. Representative images from Ponceau S staining of parasite lysates following electrophoresis and transfer to membrane, and the corresponding chemiluminescent detection of GFP with specific antibodies are shown in part F. The pre-stained marker lane is marked M above the Ponceau S panel, and the sizes of two marker proteins are indicated.

The ribozyme sequences are positioned upstream of the *Plasmodium berghei dihydrofolate reductase-thymidylate synthase* 3′-transcription terminator sequence (PbDT-3′). The resulting plasmids pGFP_*glmS* and pGFP_M9 express GFP with a mitochondrial transit peptide from *P. falciparum* heat shock protein 60. This open reading frame is under the control of 5′ and 3′ flanking sequences of *P. falciparum* heat shock protein 86 (Pfhsp86 5′) and PbDT-3′, respectively. The *P. falciparum dihydrofolate reductase-thymidylate synthase* (*Pf*DHFR-TS) gene was amplified by PCR from a previously described plasmid [24] using oligonucleotide primers dhfr-F and dhfr-R and cloned into pGFP_*glmS* and pGFP_M9 via the unique *Bgl*II and *Kpn*I sites. The resulting plasmids pDHFR-TS-GFP_*glmS* and pDHFR-TS-GFP_M9 for study of episomal reporter gene activity (Fig. 2 and 3) contain a *Pf*DHFR-TS-GFP open reading frame under the control of Pfhsp86 5′, *glmS* ribozyme and PbDT-3′ flanking sequences. The pJRTS_GFP_*glmS* plasmid for integration at the endogenous *P. falciparum* PF3D7_0417200 locus encoding *Pf*DHFR-TS was made by restriction digestion of pDHFR-TS_GFP_*glmS* with *Spe*I, followed by re-ligation of the plasmid backbone.

### Parasite culture and transfection

*Plasmodium falciparum* malaria parasites, strain 3D7 were cultured *in vitro* in human O+ erythrocytes (5% haematocrit) in RPMI-1640 medium (Invitrogen) supplemented with 0.3 g/l L-glutamine, 5 g/l hypoxanthine and 10% pooled human serum under an atmosphere of 1% O_2_ and 5% CO_2_ as described previously [25]. Pooled human serum was heat inactivated at 56°C for 45 min prior to use in culture. Ring-stage synchronized parasites were obtained by two consecutive sorbitol treatments as described in [26]. Parasite transfection was performed as described in [27]. Transfectants were obtained by selection with blasticidin at 2 μg/ml. Blasticidin-resistant transgenic parasites were obtained after 2–3 weeks of selection. Integration of the pJRTS_GFP_*glmS* plasmid was achieved by two blasticidin on/off cycles of two weeks duration each, followed by parasite cloning using the limiting dilution method in a 96-well microtiter plate. The presence of integrated transgenic DNA in blasticidin selected parasites was determined by PCR from total parasite genomic DNA using KAPA HiFi DNA polymerase (Kapa Biosystems) with 0.3 μM of each primer and 10–30 ng of genomic DNA template in 25 μl reactions. Primer annealing was performed at 64°C for the 5IntF and 5IntR pair and at 55°C for the other pairs. Sugars for parasite treatments (Glucosamine (GlcN), fructose, (Fru) mannose (Man), mannosamine (ManN) and galactosamine (GalN)) were obtained from Sigma-Aldrich and dissolved in 1× phosphate buffered saline (1×PBS: 137 mM NaCl, 2.7 mM KCl, 8 mM Na_2_HPO_4_, 2 mM KH_2_PO_4_, pH 7.4).

### RNA experiments

Synchronized ring stage parasites were treated for 24 h in the presence of 10 mM GlcN, 10 mM Fru or 1× PBS (untreated control). Parasites were harvested (typically yielding approximately 10% trophozoites) and liberated from the erythrocyte host cells by saponin lysis (0.15% w/v saponin in 1× PBS). Total RNA was extracted from the parasites using TRizol reagent following the manufacturer's recommendations (Invitrogen). Genomic DNA was removed using a Turbo DNA-*free*™kit (Applied Biosystems). RNA samples were qualified by Nanodrop (A260∶280 >2∶1) and 1% denaturing agarose gel electrophoresis (prominent 28S and 18S rRNA bands in a relative ratio 1∶1 or greater).

### RNase protection assay

Riboprobe template was synthesized by PCR amplification from pGFP_*glmS* plasmid with oligonucleotide primers glmSF and glmSR-T7 (Table S1). The PCR product was gel-purified using a QIAGEN MinElute kit. Labeled antisense riboprobe corresponding to 218 nt of the GFP_*glmS* region (Fig. 2A) was synthesized by *in vitro* transcription with T7 RNA polymerase and 0.2 mM biotin-16-UTP as recommended by the manufacturer (Fermentas). DNA template was removed from the probe by DNaseI digestion using a Turbo DNA-*free*™ kit. Total parasite RNA (20 µg) was isolated from reporter_*glmS* transgenic parasite expressing reporter gene and 3D7 parental parasites which had been treated with 10 mM GlcN for 24 h. The purified RNA was incubated with approximately 15 ng of biotin-labeled probe overnight at 42°C using an RPAIII^™^ kit (Applied Biosystems). RNase digestion was performed with 1∶1000 dilution of RNaseA/T1 enzyme from the kit for 15 min at 37°C. Purified, denatured samples were separated in a 6% Novex^®^TBE-Urea gel (Invitrogen). The separated RNA samples were transferred to a Biodyne^®^ PLUS membrane (Pall) by electrotransfer in 0.5× TBE running buffer (Invitrogen) and biotin-labeled RNA species were detected using a Phototope^®^-Star detection kit (New England Biolabs).

### Reporter gene expression study by reverse-transcription quantitative PCR (RT-qPCR)

DNA-free RNA samples (1 µg each) were reverse-transcribed with oligo dT(_21_) in 20 µl reactions at 50°C for 1 h using SuperScriptIII enzyme as recommended by the manufacturer (Invitrogen). Control reverse-transcription (RT) reactions lacking RT enzyme (-RT) were performed alongside the +RT reactions. The RT reactions were diluted 1∶100 in nuclease-free water. qPCR was performed using SsoFast^™^ EvaGreen^®^ Supermix (Bio-Rad) in 20 µl reactions. Each reaction contained 1× Supermix, 1 µl of diluted cDNA template and 0.5 µM of each oligonucleotide primer. The qPCR program used was: 95 °C for 3 min, followed by 40 cycles of 95°C 15 s, 55°C 20 s and 72°C 30 s in an iQ5 thermal cycler (Bio-Rad). PCR product threshold cycles (Cq) were calculated automatically by the iCycler program (Bio-Rad) using the default settings. In addition to –RT controls, a no-template control was performed for both primer pairs in all experiments. No product was detected (no reported Cq) in these controls for any experiment. Single amplicons of the expected size were observed for both primer pairs by thermal melt assay and 2% agarose gel electrophoresis. Under the conditions used, the primer pair efficiencies are 2.01 and 2.02 for the *glmS* and BSD pair respectively (Fig. S1). Relative changes in gene expression were calculated using the ΔΔCq method [28].

### Western immunoblot

Transgenic *P. falciparum* parasites expressing DHFR-TS-GFP (reporter_*glmS,* reporter_M9 and DHFR-TS-GFP_*glmS* integrant) were cultured in 100 ml and synchronized to 8–10% ring-stages. The culture was divided equally into four plates and GlcN added to each (10 mM final). Parasites were harvested immediately from one plate (0 h time point), and the rest were placed in culture. Parasites were harvested after 12 and 24 h of GlcN treatment. The medium was removed from the fourth plate and 75 ml of fresh medium lacking GlcN was added. The sub-cultured parasites (∼2–3% parasitemia) were divided into three plates and cultures continued for another 24 h. Parasites were harvested after 12 and 24 h culture in the fresh medium (36 and 48 h time points). Parasitized cells were harvested from each sample by centrifugation. Parasites were liberated from erythrocytes by saponin lysis and resuspended in deionised water containing protease inhibitors (0.7 µM Pepstatin (Roche) and 1× Complete EDTA-free protease inhibitor cocktail (Roche)). Proteins were extracted by freeze-thawing. Fifteen micrograms of soluble parasite protein sample (determined by Bio-Rad Bradford assay) were denatured in NuPAGE 1× LDS buffer and NuPAGE 1× reducing agent for 20–30 min at 70°C before electrophoresis in NuPAGE Novex 4–12% Bis-Tris gel with NuPAGE MOPS SDS running buffer (Invitrogen). The proteins were transferred onto nitrocellulose membrane (Protran BA85, 0.45 µm, Whatman) by electro-transfer (30 V constant for 90 min) in NuPAGE transfer buffer using a XCell II blot module (Invitrogen). The membrane was stained with 0.1% Ponceau S in 5% acetic acid for 2 min and de-stained in deionized water. The Ponceau S-stained membrane was scanned using a flat-bed scanner and densitometric lane analysis for normalization of sample loading and transfer variation was performed using NIH ImageJ software. Membranes were blocked for one hour in 5% (w/v) skimmed milk in TBST (10 mM Tris-HCl pH 8.0, 150 mM NaCl and 0.05% Tween 20). DHFR-TS-GFP protein was detected using an anti-GFP epitope tag polyclonal antibody (Thermo Scientific #PA1-19431) primary antibody diluted 1∶1000 in 5% skimmed milk/TBST and a peroxidase labeled goat anti- rabbit IgG secondary antibody (Vectorshield # Q0506) diluted 1∶10 000 in 5% skimmed milk/TBST. Bound antibody was detected using a SuperSignal West Pico chemiluminescence kit (Thermo Scientific). Measurement of DHFR-TS-GFP protein expression was performed using a ChemiDoc™ XRS+ imaging system (Bio-Rad).

### Flow cytometry analysis

Transgenic *P. falciparum* expressing DHFR-TS-GFP protein (reporter_*glmS*, reporter_M9 and DHFR-TS-GFP_*glmS* integrant) were cultured and synchronized to 1% ring form with fresh human red blood cells at 2% haematocrit. 0.09 ml of cultured parasites were transferred to individual wells of a standard 96-well microtiter plate and *in vitro* culture continued for 24 h with 0.01 ml of sugar at different concentrations in each well. The parasitized erythrocytes were analyzed using a flow cytometer (Cytomics FC 500 MPL, Beckman Coulter) equipped with a 488 nm laser. 50,000 cells were sorted per sample. The CXP program (Beckman Coulter) was used to analyze the data obtained. To determine what proportion of parasitized cells expressed GFP above background, a 525/40-nm band-pass filter (FL1) was employed with a gate threshold of 10^0^ arbitrary units. This threshold was determined from control experiments human erythrocytes infected with non-GFP expressing 3D7 *P. falciparum* (Fig. S2). To determine parasite viability, 0.1 ml of parasitized erythrocyte culture was incubated with 0.1 ml of 10 µg/ml hydroethidine (HE, Invitrogen) for 20 min at 37°C in the dark. The HE-stained parasitized erythrocytes were then analyzed using a 620/25 nm band-pass filter. The numbers of GFP-expressing or viable parasites were normalized to untreated control parasite samples in the same experiment, which were taken as 100%.

### Anti-malarial drug inhibition assays

Transgenic *P. falciparum* DHFR-TS-GFP_*glmS* integrant or wild-type 3D7 parasites were tested for sensitivity to pyrimethamine and chloroquine antimalarial drugs. 0.09 ml of cultured ring-stage synchronized parasites were transferred to individual wells of a standard 96-well microtiter plate as described above and *in vitro* culture continued for 48 h, with 0.01 ml of anti-malarial drug at different concentrations in each well. SYBR Green I was then added to each well and parasitized cells counted by flow cytometry as described above. For experiments with GlcN, 2.5 mM GlcN was added to all wells (drug and control untreated). The SYBR Green I signals in drug-treated samples were normalized to untreated control parasite samples in the same experiment, which were taken as 100%. IC_50_ values with 95% C.I. were calculated from four independent experiments.

### RNA-Seq

Transgenic DHFR-TS-GFP_*glmS* and 3D7 wild-type parasites were cultured and synchronized *in vitro* as described above. Ring-stage parasites were treated for 24 h in the presence of 10 mM GlcN or 1× PBS. Total RNA was obtained from the parasites as described above and 10–20 µg from each sample was submitted to BGI-Hong Kong for RNA-Seq paired-end transcriptome sequencing on a single lane of an Illumina HiSeq2000 101PE platform. Poor quality reads were removed from the data (reads with adaptors, >10% of bases with no call, and >50% base calls with Phred score <5) and the remaining acceptable reads were mapped to the *P. falciparum* 3D7 version 3 genome with the SOAP2 program [29]. From the mapped reads, alternative splicing events were analyzed using the MATS algorithm [30] using the default parameters, in which all pairwise combinations of samples were tested. Differential gene expression was determined using the edgeR algorithm [31]. Genes with fewer than five mapped reads in any replicate were excluded, leaving a total of 2316 (DHFR-TS-GFP_*glmS* transgenic line) and 2285 (3D7 wild-type) annotated genes for analysis. The trimmed mean of M values (TMM) was determined separately in each sample group and used to calculate normalization factors. The raw data and the results from MATS and edgeR analyses have been submitted to the NCBI GEO database under series accession number GSE43125.

### Statistical analyses

Dose-response curves were fitted to normalized cell count data from all experiments combined using the sigmoidal dose response function with variable slope in Prism v4.0 (GraphPad Software Inc.). Hill coefficient, Effective Concentration of ligand producing 50% of maximum response (EC_50_) and half-maximal inhibitory concentration (IC_50_) values were calculated without weighting. The maximum and minimum responses were constrained at 100 and 0% respectively. One sample two-tailed *t*-tests and extra sum-of-squares F- tests were performed using Prism v4.0 and the *P*-value for significance was 0.05. A False Discovery Rate (FDR) adjusted *P*-value threshold of 0.05 was used for significance in RNA-Seq data analyses.

## Results

### Testing *glmS* ribozyme function in *P. falciparum*

The potential for *glmS* ribozyme-mediated control of *P. falciparum* gene expression was first tested using an episomally expressed reporter gene of *P. falciparum* dihydrofolate reductase-thymidylate synthase (*Pf*DHFR-TS) fused to C-terminal GFP. Wild-type *glmS* ribozyme was appended to the flanking 3′ UTR downstream of the open reading frame (Fig. 2A). RNase protection assay was performed with an antisense RNA probe spanning the ribozyme cleavage site. From this experiment, RNA species corresponding to ribozyme-cleaved RNA were detected from transfected parasites expressing reporter gene-ribozyme RNA, indicating that the ribozyme was expressed as an RNA and underwent cleavage as expected (Fig. 2B). The level of reporter mRNA quantified by reverse transcription quantitative PCR (RT-qPCR) was found to be significantly reduced approximately three-fold in reporter*_glmS* parasites exposed to 10 mM GlcN for 24 h, but was unchanged in response to 24 h treatment with 10 mM Fru (Fig. 2C). In contrast, no change in reporter mRNA was observed after either treatment in transfected parasites expressing the same reporter gene, but carrying a *glmS* ribozyme with inactivating mutations at the ribozyme cleavage site (reporter_M9 parasites).

Next, in order to determine the effect of ribozyme control of gene expression at the protein level, parasitized erythrocytes expressing reporter genes treated with different sugars for 24 h were enumerated by flow cytometry. The reporter_*glmS* gene activity diminished markedly in response to GlcN (EC_50_ = 2 mM, 95% C.I. 1.7 and 2.5 mM), with much weaker attenuation using other sugars including Fru, Man, ManN and GalN (Fig. 3A). The reporter_M9 parasites expressing reporter with the mutated ribozyme did not respond to any sugar treatment (Fig. 3B), thus demonstrating the specificity of the inducible system. The markedly different response to 10 mM GlcN between reporter_*glmS* and reporter_M9 parasites was also apparent by microscopy (Fig. S3). Parasite viability, as assessed by enumerating hydroethidine stained parasites, was unaffected by these treatments (Fig. 3C, D). In order to test how quickly the ribozyme-attenuated reporter protein can recover after GlcN is withdrawn, western immunoblotting experiments were performed on reporter*_glmS* and reporter_M9 parasites cultured over one 48 h cell cycle (Fig. 3E, F). In the presence of 10 mM GlcN, reporter protein was efficiently knocked down approximately 10 fold after 24 h treatment in the reporter*_glmS* parasites. Twelve hours after GlcN was withdrawn (36 h time point), the reporter protein recovered to a level comparable to that of the reporter_M9 control. These results demonstrate that the *glmS* ribozyme can be used to attenuate gene expression in a consistent and temporal manner in *P. falciparum* by means of GlcN-induced ribozyme cleavage.

### Reverse-genetics using the *glmS* ribozyme in *P. falciparum*

**Figure 4:**
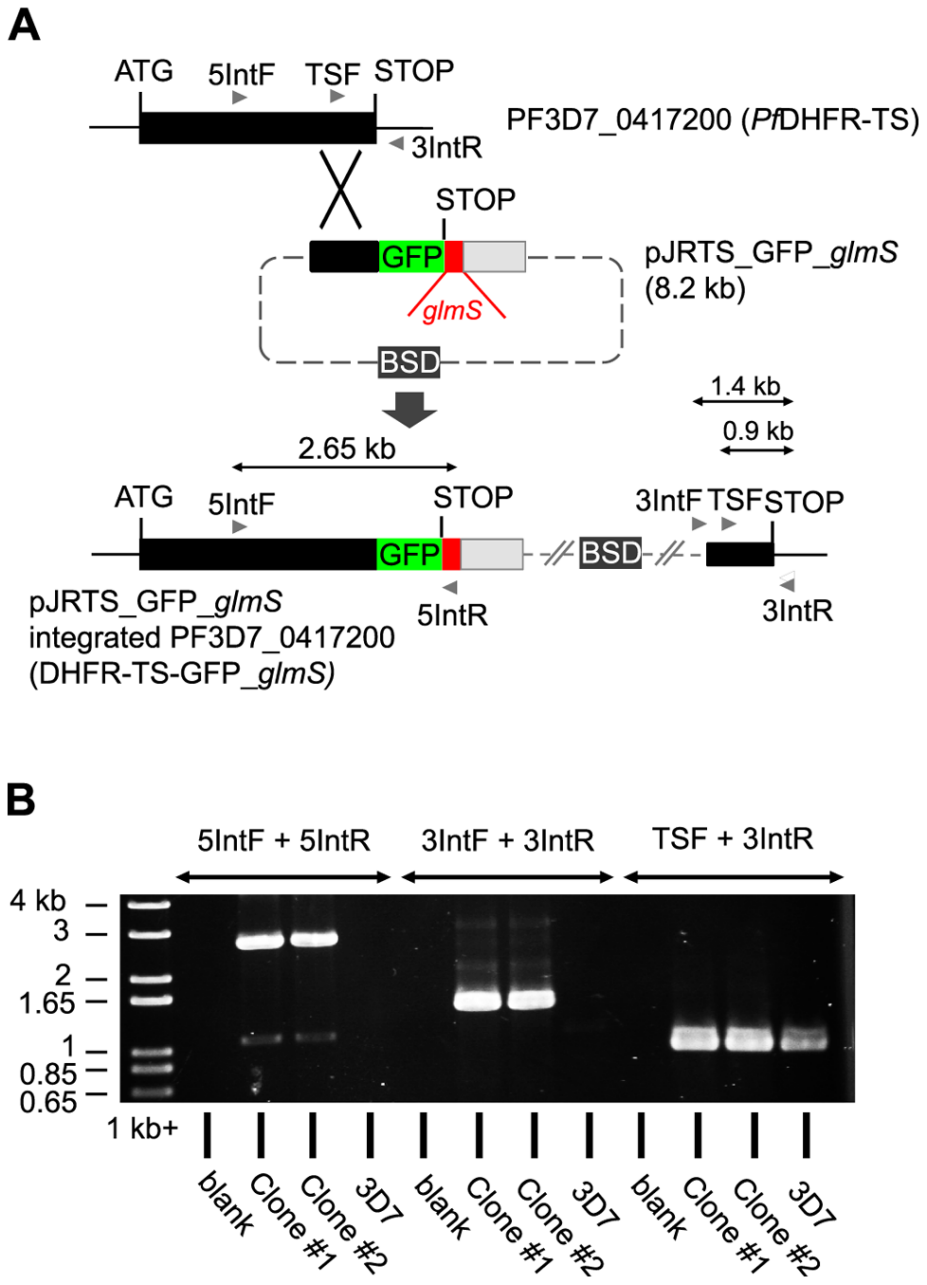
Integration of *glmS* ribozyme sequence into the essential *Pf*DHFR-TS gene. (A) Schematic showing the strategy used to incorporate the GFP and *glmS* ribozyme sequences at the 3′ end of the *Pf*DHFR-TS gene by single crossover homologous recombination. The expected sizes of PCR products shown in part B are marked. (B) PCR testing of plasmid integration from two cloned transgenic lines, clone #1 and #2. PCR primer combinations used are indicated above the lanes and the migrations of 1 kb+ DNA marker (Invitrogen) bands are indicated on the left.

**Figure 5:**
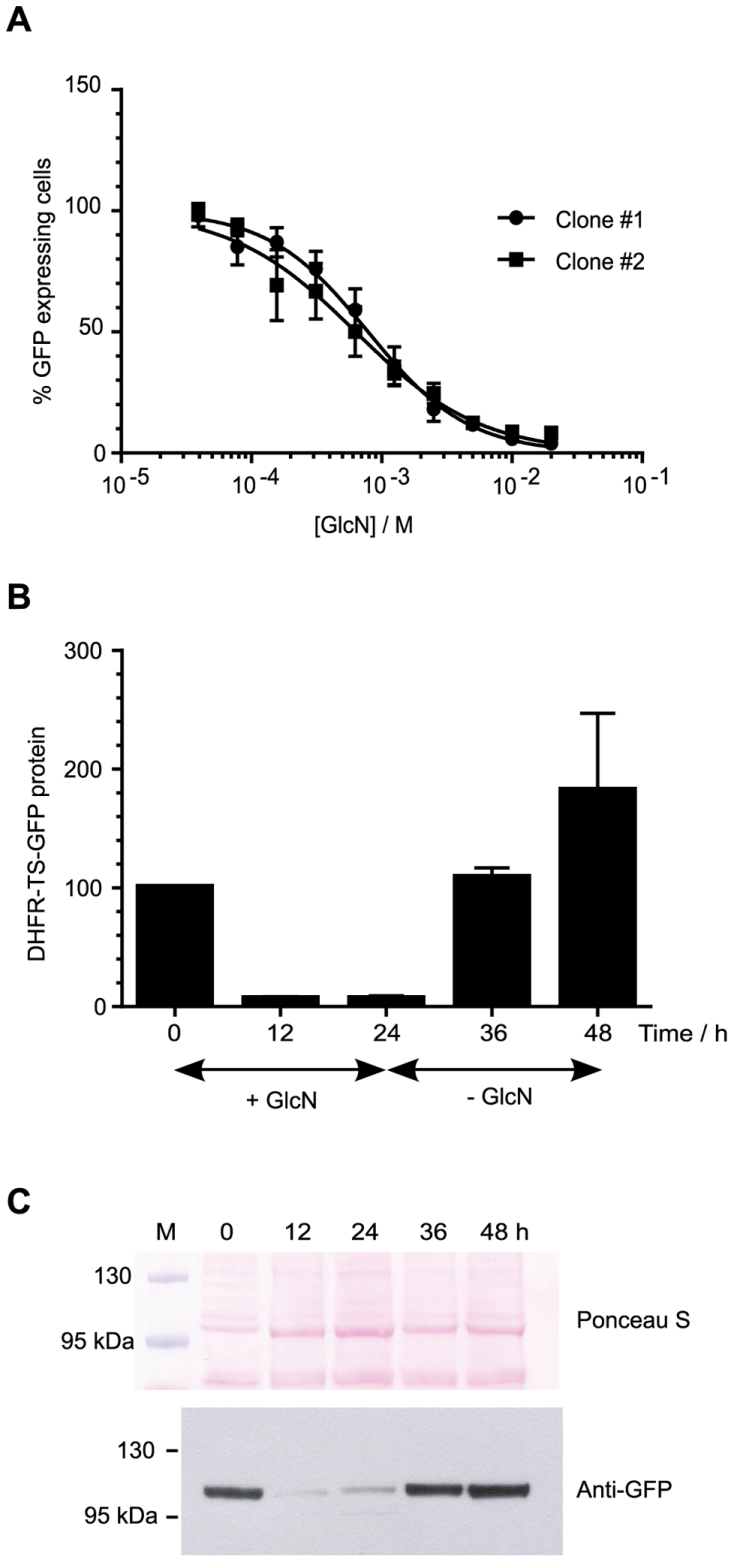
Ribozyme-mediated control of endogenous *Pf*DHFR-TS expression. (A) Knockdown of *Pf*DHFR-TS expression in DHFR-TS-GFP_*glmS* integrant parasite clonal lines in response to GlcN. Parasitized erythrocytes expressing *Pf*DHFR-TS-GFP were enumerated by flow cytometry based on the level of GFP fusion partner. Extra sum-of-squares F- test comparing individual curve fits with the null hypothesis that slope and EC_50_ are the same for both clone #1 and #2, *P* = 0.19. (B) Ribozyme-mediated knockdown of DHFR-TS-GFP protein is reversible. DHFR-TS-GFP_*glmS* integrant parasite clone #1 was cultured and treated with GlcN, and western immunoblotting to quantify DHFR-TS-GFP protein was performed as described in Fig. 3. Data are the mean from triplicate experiments and error bars represent S.E.M. (C) Representative images from Ponceau S staining of parasite lysates following electrophoresis and transfer to membrane, and chemiluminescent detection of GFP using specific antibodies. The pre-stained marker lane is marked M above the Ponceau S panel, and the sizes of two marker proteins are indicated.

To assess the potential for reverse genetics using the *glmS* ribozyme, the ribozyme sequence was integrated into the locus PF3D7_0417200 (Fig. 4A). This gene encodes the essential bifunctional enzyme *Pf*DHFR-TS, which is the target of antifolate anti-malarial drugs, including pyrimethamine and the recently developed compound P218 [32]. To facilitate monitoring of DHFR-TS expression, GFP was also fused to the C-terminal end to make a DHFR-TS-GFP fusion protein in the transgenic parasite. Two clonal lines of parasites with the expected integration were obtained (Fig. 4B). Addition of GlcN to the integrant parasite culture medium led to dose-dependent decrease in DHFR-TS-GFP protein expression within 24 h, as shown by flow-cytometric enumeration of GFP positive parasites (Fig. 5A). The GlcN response of the clone #1 showed an EC_50_ of 0.8 mM (95% C.I. 0.6 and 1.0 mM), which was not significantly different from that of clone #2. All further experiments were performed on clone #1. Western immunoblotting showed that the attenuation of DHFR-TS-GFP in the integrant was also reversible as withdrawal of GlcN led to rapid recovery of protein expression (Fig. 5B, C).

In order to validate the *glmS* ribozyme as a specific reverse-genetic tool, parasite transcriptomes after 24 h treatment with or without 10 mM GlcN were studied by RNA-Seq. Overall, parasite response to GlcN was modest with no alternative splicing events induced by GlcN treatment detected. Ten genes showed significant change in expression in the integrant and the gene showing the most significant change in expression was PF3D7_0417200 (*Pf*DHFR-TS), which was reduced approximately three-fold (Table 1).

**Table 1:** Genes showing significant changes in expression in DHFR-TS-GFP_*glmS* integrant parasites after GlcN treatment.

Plasmodb Gene ID	Product Description	uncorrected *P-*value	FDR-adj.* P-*value	log_2_ (T/U) integrant*	log_2_ (T/U) 3D7 wild-type*
PF3D7_0417200	Bifunctional dihydrofolate reductase- thymidylate synthase (*Pf*DHFR-TS)	9.47E-25	2.22E-21	−1.81	0.92
PF3D7_0218500	Small nuclear ribonucleoprotein Sm D2 (SNRPD2)	6.65E-12	7.78E-09	1.53	0.71
PF3D7_0503400	Actin-depolymerizing factor 1 (ADF1)	3.09E-11	2.41E-08	1.27	0.30
PF3D7_0830400	Conserved *Plasmodium* protein, unknown function	2.28E-08	1.33E-05	-0.93	−0.97
PF3D7_0624500	Anaphase promoting complex subunit, putative	4.99E-07	2.34E-04	−0.89	−0.44
PF3D7_0312300	26S proteasome regulatory subunit S14, putative	4.01E-06	1.56E-03	−0.94	−0.09
PF3D7_0821800	Secretory complex protein 61 beta subunit (Sec61-beta)	2.26E-05	7.54E-03	−1.54	−1.08
PF3D7_1103700	Casein kinase II beta chain (CK2beta1)	1.52E-04	3.53E-02	−0.64	−0.06
PF3D7_1124900	60S ribosomal protein L35, putative	1.66E-04	3.53E-02	−0.62	−0.48
PF3D7_1403400	Conserved *Plasmodium* protein, unknown function	2.34E-04	4.57E-02	1.05	0.21

* Changes in gene expression are shown as the log_2_ mean normalized ratio of GlcN treated to untreated from 2 replicates.

**Figure 6:**
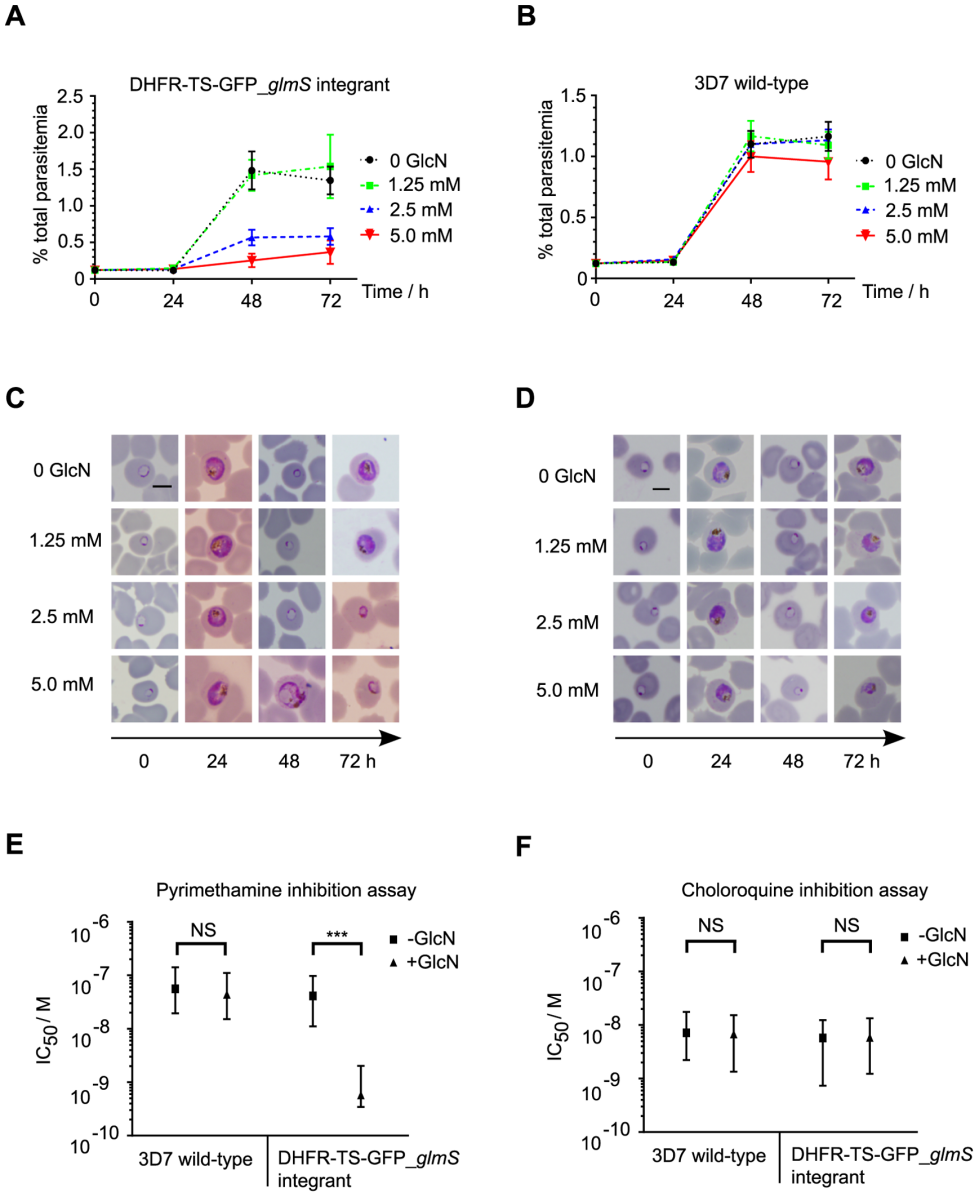
Ribozyme-mediated *Pf*DHFR-TS knockdown phenotype. Parasite growth in cultures of DHFR-TS-GFP_*glmS* integrant (A) or wild-type 3D7 (B) treated with varying levels of GlcN for up to 72 h was determined by counting 1000 infected erythrocytes from Giemsa-stained slides for each treatment time point. The data are the mean from triplicate experiments and error bars represent S.E.M. The morphology of treated parasites at different time points of treatment is shown for DHFR-TS-GFP_*glmS* integrant (C) or wild-type 3D7 (D). Panels are representative images from Giemsa-stained slides. Scale bars, 5 μm. Anti-malarial drug inhibition assays for pyrimethamine (E) and chloroquine (F) were performed in the presence (+ GlcN) or absence (-GlcN) of 2.5 mM GlcN in the parasite culture. The IC_50_ values shown are the fitted values from four independent experiments and error bars represent the 95% C.I. Extra sum-of-squares F-test *P*-values comparing individual curve fits with the null hypothesis that slope and IC_50_ are the same for both + GlcN and – GlcN: DHFR-TS-GFP_*glmS* integrant pyrimethamine *P*<0.0001; wild-type 3D7 pyrimethamine *P* = 0.7138; DHFR-TS-GFP_*glmS* integrant chloroquine *P* = 0.4554; wild-type 3D7 chloroquine *P* = 0.7898.

Next, longer treatments with GlcN were performed to test whether they would lead to a loss-of-function phenotype in the integrant parasite. A marked GlcN dose-dependent reduction of integrant parasite development and reinvasion of erythrocytes was observed after one growth cycle (Fig. 6A). In contrast, slight growth retardation was observed for wild–type 3D7 parasites only at 5 mM GlcN (Fig. 6B). Abnormal transgenic DHFR-TS-GFP *glmS* parasite morphology was observed already after 48 h exposure to 5 mM GlcN, and the majority of parasites in the next cycle at 72 h showed evidence of further delay and abnormal development in the 2.5 and 5 mM GlcN treatments (Fig. 6C). Among a minority of wild–type 3D7 parasites, abnormal morphology (slightly shrunken trophozoites) was observed only after 72 h treatment with 5 mM GlcN (Fig. 6D). In addition to a growth defect, GlcN-induced reduction of DHFR-TS-GFP protein in integrant parasites sensitized them to pyrimethamine, but not chloroquine, a drug targeting heme crystallization. GlcN had no effect on the wild–type 3D7 parasite sensitivity to either drug (Fig. 6E, F).

## Discussion

We have demonstrated that the *glmS* ribozyme is active in *P. falciparum* and can be used as a tool to modulate target gene expression in transgenic parasites. Reverse-genetics to understand the function of essential parasite genes is possible with the *glmS* ribozyme tool without the requirement for promoter modification or protein fusion to destabilizing domains. The level of *P. falciparum* target protein knockdown achievable is comparable to the previously described DD inducible system. On the other hand, the *glmS* ribozyme and DD systems could be combined to provide an even greater control of target protein, provided that the DD does not interfere with the target protein function.

The RNA experiments in Fig. 2 demonstrated *glmS* ribozyme cleavage of target *P. falciparum* mRNA. The three-fold reduction of mRNA after induction of ribozyme cleavage can be attributed to mRNA degradation by the cytoplasmic 3′ exosome machinery, as described in *Saccharomyces cerevisiae* yeast [33]. In that work, it was shown that the ribozyme-cleaved mRNA 5′ fragment separated from its polyA tail is rapidly degraded in wild-type yeast, but is stable in *ski7* mutant yeast defective in 3′ exosome function. In all our experiments, the *glmS* ribozyme was positioned in the 3′ UTR as this facilitates single-crossover integration at target gene loci. The *glmS* ribozyme may also function efficiently in the 5′ UTR position in a similar fashion to the Sm1 hammerhead ribozyme, as shown in *P. falciparum* [20]. Moreover, positioning of the *glmS* ribozyme in the 5′ UTR may be more efficient than in the 3′ UTR. This is because mRNA cleavage in the 5′ UTR would separate the mRNA from its 5′ cap structure. The de-capped mRNA would then be susceptible to degradation by the cytoplasmic 5′–3′ exonclease XRN-1, which acts more rapidly than the 3′ exosome acting on de-adenylated mRNA [33].

One caveat of the ribozyme system, in common with any other inducible system, is the confounding effect of the inducing ligand by itself. Although prolonged GlcN treatment at high dose is toxic to parasites [34], the effective range of GlcN concentration for *glmS* ribozyme mediated knockdown is below the level of toxicity causing cell death and gross morphological change. On the other hand, it is possible that GlcN toxicity may confound phenotypic analysis of essential gene knockdowns at certain stages of parasite development. In these circumstances, the inactive ribozyme mutant M9 would be a useful control. From RNA-Seq, some genes are down-regulated in response to GlcN in the wild-type 3D7 parasite (Table S2). For these genes, the confounding effect of GlcN on expression in the control parasite may interfere with phenotypic analysis of the knocked-down transgenic parasite. To overcome this limitation, other metabolite controlled ribozymes such as the theophylline-inducible aptazyme [35] warrant further investigation as reverse-genetic tools.

The *glmS* ribozyme-mediated knockdown of *Pf*DHFR-TS led to a growth defect, consistent with the accepted notion that this gene is essential in *P. falciparum*. Although *Pf*DHFR-TS knockdown is detectable after 12 h GlcN treatment (Fig. 5B), the overall transcriptional response of the parasite is very limited and morphological changes are not observed until after 48 h of treatment. Lethal concentrations of antifolates targeting *Pf*DHFR-TS also elicit a weak transcriptional response, and morphological defects are not observable until 24 h of drug exposure [36]. In addition to a knockdown phenotype, the *glmS* ribozyme-mediated knockdown of *Pf*DHFR-TS hypersensitized the parasite to pyrimethamine. This result is consistent with *Pf*DHFR-TS as the target of pyrimethamine using the same logic that yeast haploinsufficient for DFR1 encoding dihydrofolate reductase are hypersensitive to methotrexate, a potent antifolate drug [37]. Likewise, it can be envisioned that a collection of transgenic parasites with *glmS* ribozyme integrated at different essential genes could be used to systematically identify anti-malarial compound mode of action and accelerate drug discovery against this important pathogen. We caution though that further testing of the *glmS* ribozyme tool in other gene targets is required in order to determine how generally useful this strategy is. It is not known whether genes expressed more abundantly than *Pf*DHFR-TS, or expressed in a different pattern throughout the life cycle could be efficiently regulated by the *glmS* ribozyme tool.

Furthermore, since the *glmS* ribozyme is active when expressed in the 3′-UTR of target genes, it could be applied easily for knockdown of essential genes in other parasite species amenable to transfection and homologous recombination, even those species for which little is known of gene regulation such as *Eimeria sp*. and *Babesia bovis*.

## Supporting Information

Figure S1 **RT-qPCR assay validation.** (A) Determination of qPCR primer pair efficiencies by amplification of cDNA dilutions. Each point is the mean value from triplicate experiments. Primer efficiency was calculated from the slope of the best-fit linear regression. (B) Expression of the normalizing gene BSD does not change in response to sugar treatment. The change in BSD expression in parasites treated with 10 mM sugar for 24 h compared with untreated parasites was calculated using the 2^−(Cq treated-Cq untreated)^ transformation; error bars represent S.E.M. To test the null hypothesis that treatment does not cause a change in BSD expression, two-tailed one-sample *t*-tests comparing sample mean with hypothetical mean = 1 were performed. The calculated *P*-values are 0.9046, 0.0811, 0.36, 0.605 for reporter_*glmS* GlcN, reporter_M9 GlcN, reporter_*glmS* Fru, and reporter_M9 Fru respectively.
(TIF)

Figure S2 **Flow cytometry analysis of GFP expressing cells.** Representative raw data scatter-plots from flow cytometry experiments with *P. falciparum* infected human erythrocytes are shown. Control non-GFP expressing 3D7 wild-type parasite (untreated) is shown in part A. Transgenic parasites are shown in (B) reporter_*glmS* (untreated), (C) reporter_M9 (untreated), (D) reporter_*glmS* (10 mM GlcN treated) and (E) reporter_M9 (10 mM GlcN treated). The FL1 threshold of 10^0^ (arrowed) was chosen for gating GFP-positive cells, since no cells were counted as positive above this threshold in control non-GFP expressing parasites.
(TIF)

Figure S3 **Fluorescence microscopy of live parasites.** Representative overlaid GFP/bright-field images from (A) reporter_*glmS* and (B) reporter_M9 transgenic parasites are shown for control untreated on the left, and after 24 h treatment with 10 mM GlcN on the right. Images were taken using an Olympus BX51 microscope equipped with an Olympus DP71 digital camera and DP controller software v 3.2.1.276. All images were obtained using an exposure time of 1/3.5 s with ISO sensitivity ISO200.
(TIF)

Table S1 Sequences of oligonucleotides used in this study.
(DOCX)

Table S2 Genes significantly down-regulated in wild-type 3D7 *P. falciparum* following treatment with 10 mM GlcN for 24 h.
(XLSX)
