# Supplementary figures and images for: Inducible Knockdown of Plasmodium Gene Expression Using the glmS Ribozyme
Source: PLoS One. 2013 Aug 30;8(8):e73783. doi: 10.1371/journal.pone.0073783 (PMC3758297; doi:10.1371/journal.pone.0073783)

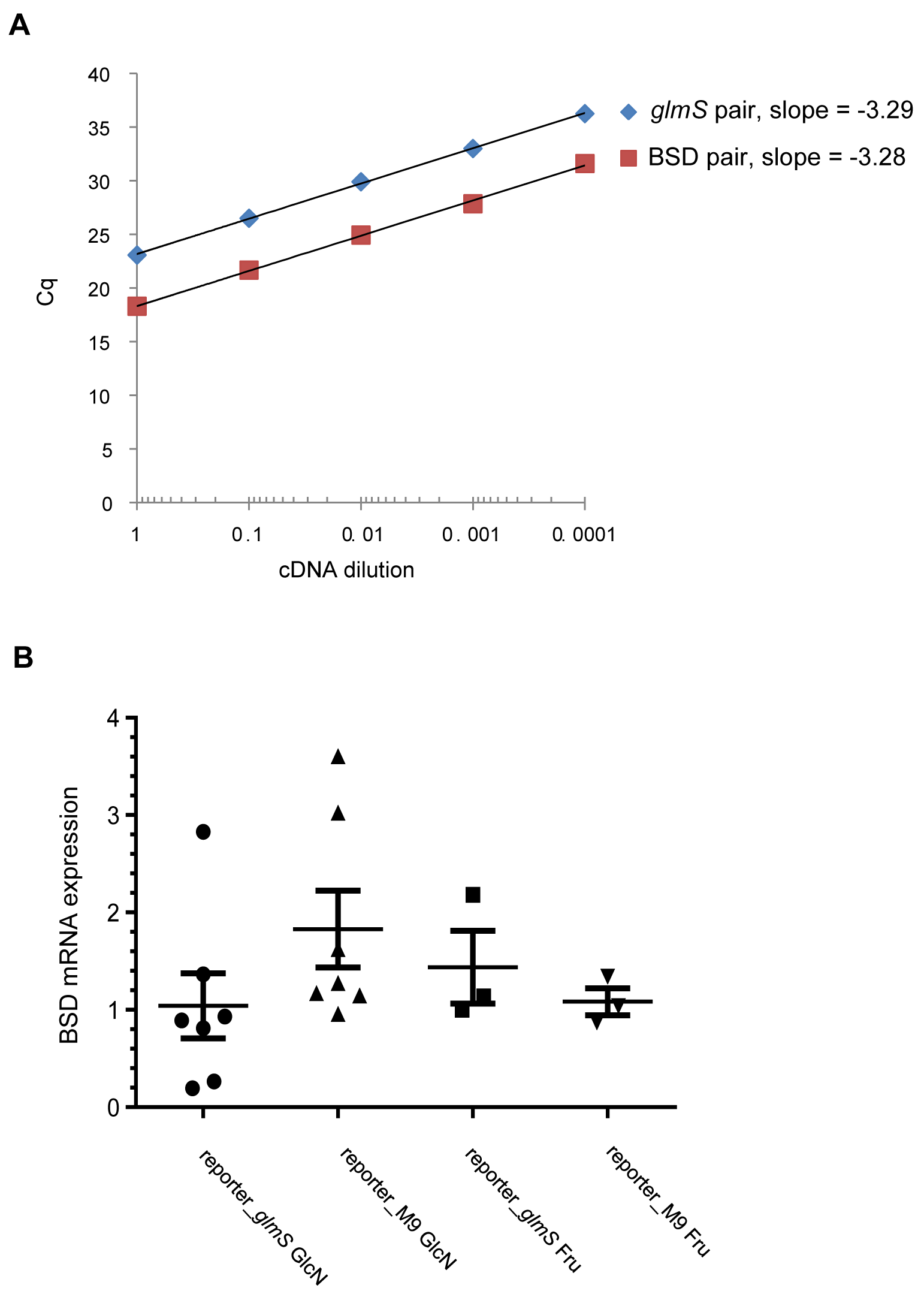

Supplement: Figure S1 — RT-qPCR assay validation. (A) Determination of qPCR primer pair efficiencies by amplification of cDNA dilutions. Each point is the mean value from triplicate experiments. Primer efficiency was calculated from the slope of the best-fit linear regression. (B) Expression of the normalizing gene BSD does not change in response to sugar treatment. The change in BSD expression in parasites treated with 10 mM sugar for 24 h compared with untreated parasites was calculated using the 2−(Cq treated-Cq untreated) transformation; error bars represent S.E.M. To test the null hypothesis that treatment does not cause a change in BSD expression, two-tailed one-sample t-tests comparing sample mean with hypothetical mean = 1 were performed. The calculated P-values are 0.9046, 0.0811, 0.36, 0.605 for reporter_glmS GlcN, reporter_M9 GlcN, reporter_glmS Fru, and reporter_M9 Fru respectively. (TIF) [file pone.0073783.s001.tif]

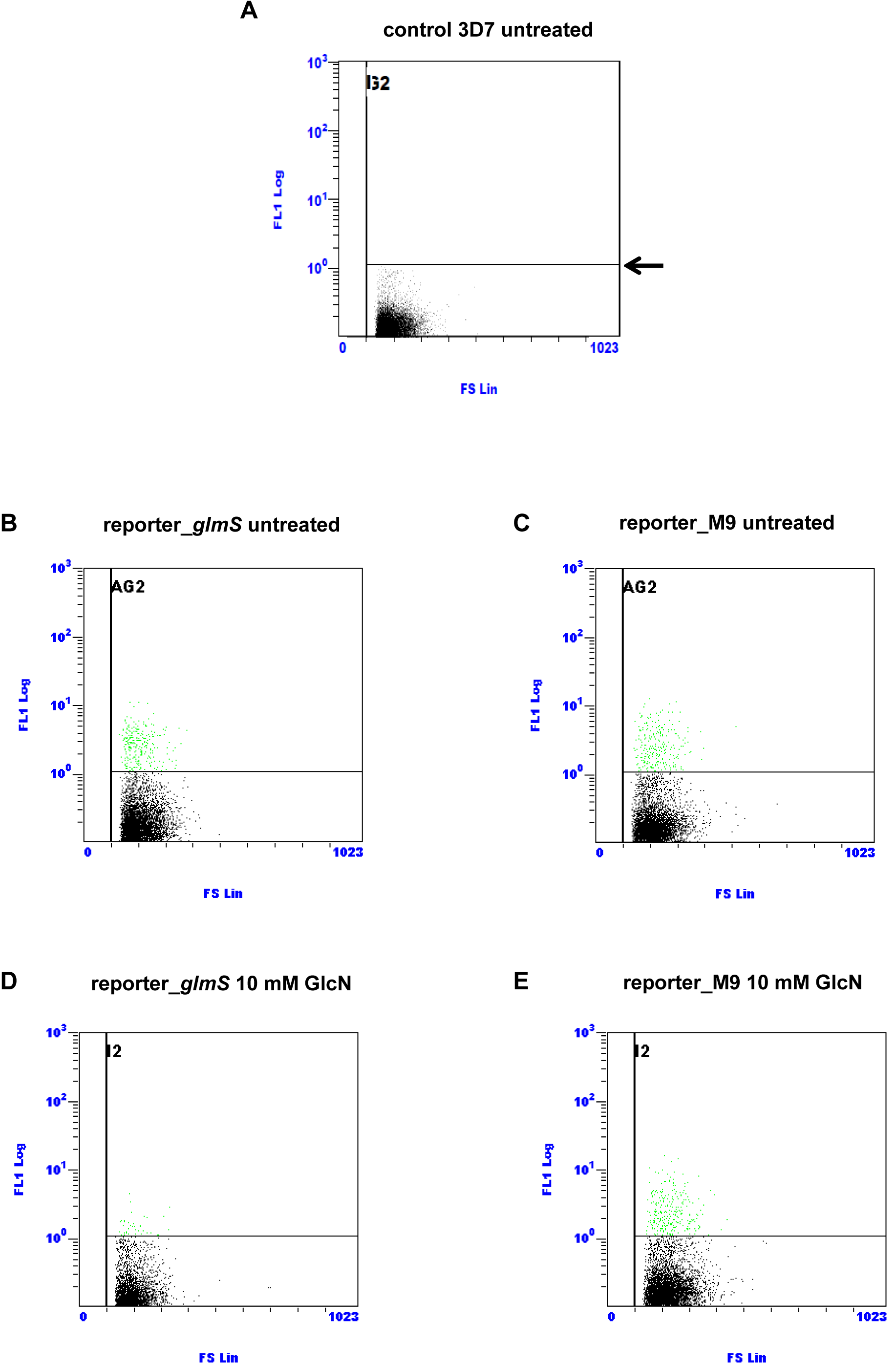

Supplement: Figure S2 — Flow cytometry analysis of GFP expressing cells. Representative raw data scatter-plots from flow cytometry experiments with P. falciparum infected human erythrocytes are shown. Control non-GFP expressing 3D7 wild-type parasite (untreated) is shown in part A. Transgenic parasites are shown in (B) reporter_glmS (untreated), (C) reporter_M9 (untreated), (D) reporter_glmS (10 mM GlcN treated) and (E) reporter_M9 (10 mM GlcN treated). The FL1 threshold of 100 (arrowed) was chosen for gating GFP-positive cells, since no cells were counted as positive above this threshold in control non-GFP expressing parasites. (TIF) [file pone.0073783.s002.tif]

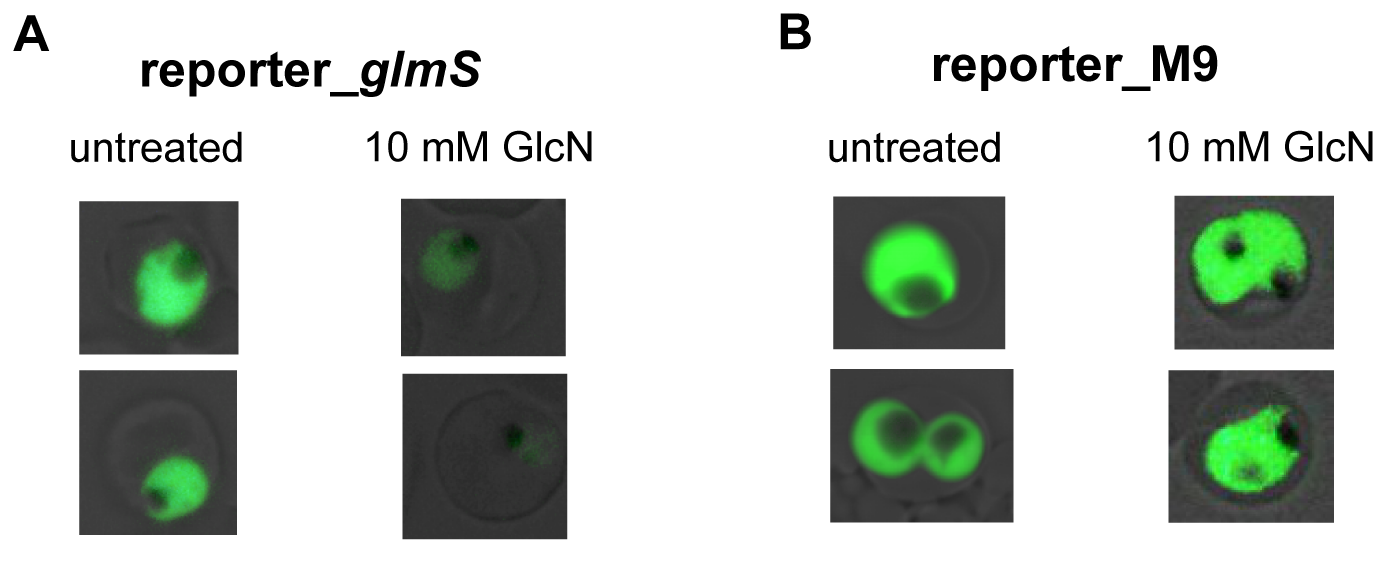

Supplement: Figure S3 — Fluorescence microscopy of live parasites. Representative overlaid GFP/bright-field images from (A) reporter_glmS and (B) reporter_M9 transgenic parasites are shown for control untreated on the left, and after 24 h treatment with 10 mM GlcN on the right. Images were taken using an Olympus BX51 microscope equipped with an Olympus DP71 digital camera and DP controller software v 3.2.1.276. All images were obtained using an exposure time of 1/3.5 s with ISO sensitivity ISO200. (TIF) [file pone.0073783.s003.tif]
