# Supplementary material for: Inducible Knockdown of Plasmodium Gene Expression Using the glmS Ribozyme
Source: PLoS One. 2013 Aug 30;8(8):e73783. doi: 10.1371/journal.pone.0073783 (PMC3758297; doi:10.1371/journal.pone.0073783)
Supplement: Table S1 — Sequences of oligonucleotides used in this study. (DOCX) [file pone.0073783.s004.docx]

| Oligonucleotide Name | Oligonucleotide sequence (5'-3') |
| --- | --- |
| syn1 | TTGGATCCCCGAACTAAGCGCCCGGAAAAAGGCTTAGTTGA |
| syn2 | GGATGGAGGTTATCGAATTTTCGGCGGATGCCTCCCGGCTGAG |
| syn3 | GATCACAGCCGTAAGGATTTCTTCAAACCAAGGGGGTGACTCCTT |
| syn4 | AATCTAGATGTGATTTCTCTTTGTTCAAGGAGTCACCCCCTT |
| syn5 | AATCCTTACGGCTGTGATCTGCACACTCAGCCGGGAGGCAT |
| syn6 | AATTCGATAACCTCCATCCTCGTCAACTAAGCCTTTTTCCG |
| syn7 | TATCTCGAGAGATCATGTGATTTCTCTTTGTTCAAGGAG |
| syn8 | TATCTGCAGTAATTATAGCGCCCGAACTAAGCGCCCGGA |
| syn9 | TATCTGCAGTAATTATCCCGCCCGAACTAAGCGCCCGGA |
| dhfr-F | AAGGATCCATGATGGAACAAGTCTGCGACGTTTTC |
| dhfr-R | AAGGTACCAGCAGCCATATCCATTGAAATTTTTTCA |
| glmSF | CGAAAGATCCCAACGAAAAG |
| glmSR-T7 | TAATACGACTCACTATAGGGGGCTGTGATCTGCACACTCA |
| BSD-F | AATAAATCATAATTGTCTCAAGAAGAATCCAC |
| BSD-R | AATAAATCATAATCCCCCAGTAAAATGATATAC |
| glmSqPCR-F | AATAAATCATAAAGTGGAGAGGGTGAAGGTGA |
| glmSqPCR-R | AATAAATCATAACTGGGTATCTCGCAAAGCAT |
| 5IntF | ATACAAGCTTATGATGGAACAAGTCTG |
| 5IntR | TCGATAACCTCCATCCTCGT |
| 3IntF | TAACTTAGGTCTCACGGTAA |
| 3IntR | AAGGTACCGTTCAGGTAATTTTGTCATC |
| TSF | GAATGGAAATAAACAAAGTG |

Supplementary Table S1. Oligonucleotides used in this study
